# P-224. Accurate *Clostridioides difficile* Ribotype Prediction from Whole Genome Sequencing Data Using Machine Learning

**DOI:** 10.1093/ofid/ofae631.428

**Published:** 2025-01-29

**Authors:** Peng Qi, Susannah McKay, Ashley Paulick, Nick Vlachos, Michelle Adamczyk, Bree E Friedman, Bonnie Weber, Amy Gargis, Alison L Halpin

**Affiliations:** CDC, Atlanta, Georgia; CDC, Atlanta, Georgia; Centers for Disease Control and Prevention, Atlanta, GA; Centers for Disease Control and Prevention, Atlanta, GA; CDC, Atlanta, Georgia; University of Minnesota, St. Paul, Minnesota; Minnesota Department of Health, Saint Paul, Minnesota; CDC, Atlanta, Georgia; Centers for Disease Control and Prevention, Atlanta, GA

## Abstract

**Background:**

*Clostridioides difficile* is a healthcare-associated pathogen recognized as the most common cause of infectious diarrhea in healthcare settings. PCR-based ribotyping, which employs heterogeneity of the ribosomal intergenic spacer region for discrimination, has historically been the preferred method for *C. difficile* typing. However, ribotyping is laborious and exhibits inter-laboratory variation which significantly limits its utility as a universal typing scheme.

The increasing adoption of whole genome sequencing (WGS) provides the opportunity for universal prediction of ribotypes (RTs) from WGS data. Machine learning (ML) offers the opportunity to predict RTs from genomic features which are more amenable to reliable resolution by WGS analysis than the repetitive sequences which define RTs.

**Methods:**

Here we employed 8 different ML models using a dataset of 2,283 *C. difficile* isolates collected through surveillance activities (2012-2018) with both CDC’s capillary-based PCR-ribotyping and whole genome multi-locus sequence type data (6,786 loci; BioNumerics v7.6). The dataset was divided into training (75%) and testing (25%) sets to train and test each model. Models were improved with hyperparameter tuning. Accuracy was calculated from the predicted RTs compared to the PCR-based RTs of the test set. We combined the top three models based on probability distribution of correct/incorrect predictions to enhance performance. The final model was applied on WGS data for 2,194 isolates collected during 2019-2020.

**Results:**

The top performing models were Support Vector Machine (linear kernel) (93.2% accuracy), Xgboost (92.6%), and Logistic Regression (92.6%). The final combined model achieved 94.3% accuracy overall with 44/51 RTs reliably predicted (99.2%). Three RTs could not be reliably predicted (64.0%); one RT could not be predicted due to the insufficient number of isolates available in the dataset. Application of the final model on the 2019-2020 dataset reliably predicted 93.2% (1816/1948) of RTs called; 11% of isolates lacked a RT call.

**Conclusion:**

Use of ML on WGS data shows promise as a universal method for accurate *C. difficile* RT prediction and thus for identifying clinically important strains while providing an important bridge to connect WGS with historical RT trends.

**Disclosures:**

**All Authors**: No reported disclosures

